# Delaunay Triangulation-Based Spatial Clustering Technique for Enhanced Adjacent Boundary Detection and Segmentation of LiDAR 3D Point Clouds

**DOI:** 10.3390/s19183926

**Published:** 2019-09-12

**Authors:** Jongwon Kim, Jeongho Cho

**Affiliations:** Department of Electrical Engineering, Soonchunhyang University, Asan 31538, Korea

**Keywords:** Delaunay triangulation, spatial clustering, point cloud, adjacent boundary

## Abstract

In spatial data with complexity, different clusters can be very contiguous, and the density of each cluster can be arbitrary and uneven. In addition, background noise that does not belong to any clusters in the data, or chain noise that connects multiple clusters may be included. This makes it difficult to separate clusters in contact with adjacent clusters, so a new approach is required to solve the nonlinear shape, irregular density, and touching problems of adjacent clusters that are common in complex spatial data clustering, as well as to improve robustness against various types of noise in spatial clusters. Accordingly, we proposed an efficient graph-based spatial clustering technique that employs Delaunay triangulation and the mechanism of DBSCAN (density-based spatial clustering of applications with noise). In the performance evaluation using simulated synthetic data as well as real 3D point clouds, the proposed method maintained better clustering and separability of neighboring clusters compared to other clustering techniques, and is expected to be of practical use in the field of spatial data mining.

## 1. Introduction

Spatial data mining, which not only intelligently extracts implicit and useful information from a large amount of spatial data, but also considers spatial autocorrelation and heterogeneity, has become increasingly necessary as the size of such data and the associated complexity increases [1]. After analyzing data without prior knowledge, such as the probability distribution or the number of clusters, classification into similar groups is a key aspect of spatial data mining. For this, spatial clustering must be initially performed. Spatial clustering considering geometric coordinate information representing spatial properties is an analytical method that divides the whole dataset into groups using the distance or similarity between the data. Spatial clustering analyzes geospatial data with different characteristics and finds meaningful patterns or regularities in spatial data [2].

Since k-means [3] was proposed in 1967, numerous studies have been carried out regarding spatial data analysis. In particular, as computer hardware has developed rapidly since the 1990s and machine learning/pattern recognition has grown steeply, spatial clustering techniques with excellent performance have begun to emerge. Among them are density-based clustering [4,5,6,7], which calculates density according to neighboring data, and graph-based clustering [8,9,10], which extracts corresponding points using a mathematical model through an affinity matrix. These techniques show excellent performance in detecting not only clusters with uniform data distribution and ideal shapes, but also clusters with some nonlinearity, and have been applied to various fields such as traffic accident analysis [11], seismic analysis [12,13], image segmentation [14], and three-dimensional object modeling [15] based on sensor information.

However, clustering methods still have limitations when faced with datasets that differ in size, shape, density, noise, and outliers [16]. Most of the spatial clustering methods proposed in previous studies are based on geometric attribute such as distance, shape, and density, which is later compared with the threshold value to determine whether it is included in the cluster. These methods are not easily applied to complex spatial data, although the results are relatively easy to interpret and the computation process is simple [17]. Within spatial data with complexity, different clusters can be very contiguous, and the density of the data forming the cluster can be anomalous and arbitrary. In addition, background noise that does not belong to any clusters in the data, or a touching issue, e.g., multiple bridges, neck problem, and adjacent problem (see Figure 2), that connects multiple clusters may be included [18]. This makes it difficult to separate adjacent clusters as one cluster can be divided into several small clusters, or all of the spatial data can be recognized as one cluster [19]. As a result, meaningful data are judged as noise or outliers, which can have a serious effect on the performance of clustering. There is now a need for a solution to the various problems arising from the complexity of spatial data.

K-means, PAM (Partitioning around medoids) [20], and CLARANS [21] when applied as a partitioning clustering method perform clustering in the direction of minimizing the distance between the spatial data and the centroid of the clusters based on the number of pre-assigned clusters. Although these methods are relatively simple and highly efficient, they are applicable only when the clusters are spherical or similar in size, which limits the spatial data clustering. BIRCH [22], as a hierarchical clustering scheme, groups individual entities sequentially or hierarchically and performs clustering based on the distance and similarity of the data—clusters with relatively different shapes and sizes can be handled by separating them according to the layers, but this approach is also vulnerable to nonlinear spatial data clustering.

**Figure 1:**
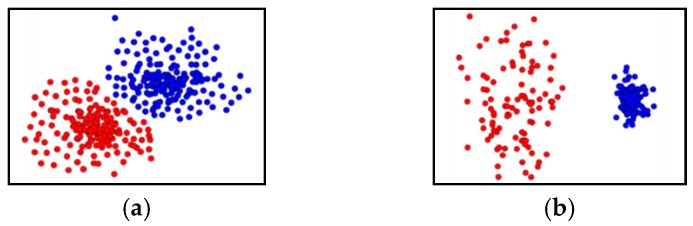
Examples of clusters with uneven densities. (**a**) represents a database with clusters of various densities; (**b**) shows clusters with uneven densities.

To overcome such limitations of nonlinear clustering through these existing methods, DBSCAN [23] was proposed to cluster based on the density of neighbors, and various density-based clustering techniques such as OPTICS [24] and DENCLUE [25] were derived. They can cluster any geometric clusters with nonlinearity to a good level and are very robust to background noise and anomalies. However, for example, if spatial data consisting of the clusters that are too close to each other or composed of irregular densities, as shown in Figure 1, this negatively impacts clustering performance.

**Figure 2:**
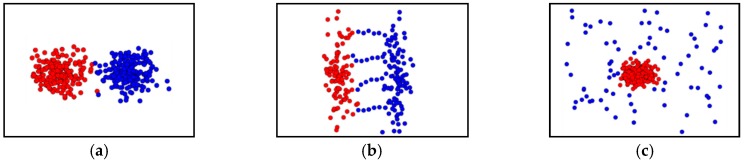
Examples of the touching problem. (**a**) the neck problem; (**b**) the chaining problem; (**c**) the adjacent problem.

In a relatively recent study, graph-based clustering techniques such as spectral clustering (SC) [26], AUTOCLUST [27], and AMOEBA [28] have been proposed to show that clusters with uneven densities can be successfully separated in spatial data, ensuring robustness against background noise. However, there are still limits due to the touching problem and chain noise, as shown in Figure 2. Although many studies have been conducted, they have had a negative impact on clustering performance due to their inherent limitations when applied to complex spatial data. In particular, density and graph-based clustering techniques are excellent for nonlinear clustering, i.e., irregular densities and noise problems that exist throughout the data. However, they are not only susceptible to noise such as with the chaining problem, but also have difficulty separating clusters when in contact with adjacent clusters.

Thus, we propose Delaunay triangulation-based spatial clustering of application with noise (DTSCAN), which can overcome low-level robustness against noise, mitigate the difficulties of separating adjacent clusters, and smoothly detect clusters of various density characteristics and geometric shapes. DTSCAN is a graph-based spatial clustering technique that employs Delaunay triangulation, which is a common tool in geometric spatial analysis, and the concept of neighboring objects is used in DBSCAN’s clustering mechanism. Although clustering techniques using triangulation have already been implemented in some studies, DTSCAN differs somewhat compared to existing techniques. The proposed strategy assumes an undirected graph consisting of vertices and edges of triangles generated by triangulation. The length of the edge and the area of a triangle are then normalized using the z-score to suppress the nodes connected to adjacent clusters and noise existing in the generated graph, and the unnecessary components are sequentially removed by selecting an appropriate threshold value. In the clustering phase, a new approach to clustering based on the density of neighbors in the graph is attempted using DBSCAN’s clustering mechanism to ensure robustness against the chaining problems and the adjacency problems of clusters.

To verify the performance of the proposed DTSCAN, virtual two-dimensional spatial data and three-dimensional point clouds from LiDAR collected by an actual driving ground vehicle were utilized. Experimental results showed that it can achieve superior clustering and separability of neighboring clusters in complex spatial data compared to existing techniques, and is expected to be of practical use in the field of spatial data mining.

## 2. Delaunay Triangulation

Triangulation applied to computational geometry divides multidimensional data consisting of polygons and polyhedral into triangles or tetrahedrons that share only the vertices of the adjacent region or only the low dimensional plane. Triangulation is treated as part of a proximity search or entity partitioning depending on the nature of the object that needs to be partitioned. Although the triangles have the same number of components, they have different properties depending on the partitioning technique such as satisfying certain properties and optimizing the objective function.

Delaunay triangulation splits the space by connecting the points on the plane with triangles so that the minimum value of the internal angles of these triangles is maximized, and each of the divided triangles is nearly an equilateral triangle [29]. The divided triangles exhibit similarity with adjacent objects, which determine the data distribution characteristics. Because of these characteristics, Delaunay triangulation is widely used not only in computer geometry, but also in computer vision, pattern recognition, and data mining.

**Figure 3:**
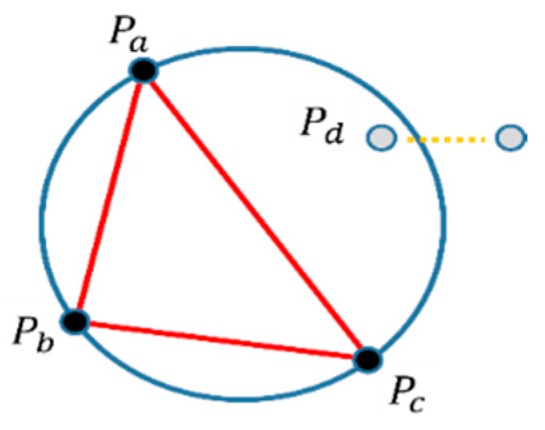
Example of the verification of Delaunay triangulation.

Given a finite N point set $Q=\left\{p_{1},\cdots ,p_{N}\right\}\subseteq R^{2}$, consisting of spatial data points of $p_{i}=\left\{x_{i},y_{i}\right\}\in Q$, its Delaunay triangulation is $DT\left(Q\right)=\left\{T_{1},\cdots ,T_{H}\right\}$. Here, T is a set of vertices of a triangle corresponding to the elements of the spatial point set, Q, and the number of triangles, H, is at most 2N−2 according to the point data distribution. Note that any point of Q should not be included in the circumscribed circles surrounding any triangle belonging to DT(Q). This verification process is at the core of the Delaunay triangulation, and various segmentation techniques are derived, depending on the data structure, dimensions, and computation speed. Let a set of arbitrary points be $Q=\left\{P_{a},P_{b},P_{c},P_{d}\right\}$ on a two-dimensional plane as shown in Figure 3. The existence of $P_{d}$ within the circumcircle of a triangle, $P_{a-c}$, can be generally detected by evaluating the determinant of Eq. (1); when the triangle is aligned in a counterclockwise order, point $P_{d}$ lies inside the triangle’s circumcircle if and only if the determinant of Equation (1) becomes positive.

(1) $$\left(\begin{array}{ccc} x_{a} & y_{a} & \begin{array}{cc} x_{a}^{2}+y_{a}^{2} & 1 \end{array}\\ x_{b} & y_{b} & \begin{array}{cc} x_{b}^{2}+y_{b}^{2} & 1 \end{array}\\ \begin{array}{c} x_{c}\\ x_{d} \end{array} & \begin{array}{c} y_{c}\\ y_{d} \end{array} & \begin{array}{c} \begin{array}{cc} x_{c}^{2}+y_{c}^{2} & 1 \end{array}\\ \begin{array}{cc} x_{d}^{2}+y_{d}^{2} & 1 \end{array} \end{array} \end{array}\right)$$

## 3. Density-Based Spatial Clustering using Delaunay Triangulation

**Figure 4:**
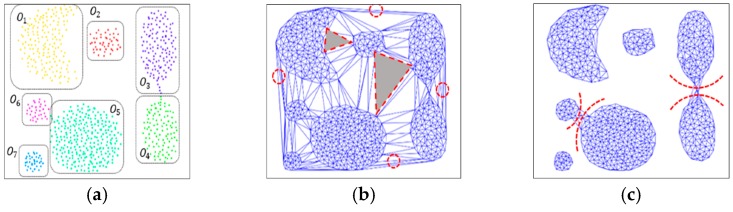
Simulated spatial data and its clustering. (**a**) original data; (**b**) Delaunay triangulation of the data; (**c**) graph after removing global effect.

DTSCAN extends all of the edges $Edges\left(DT\left(Q\right)\right)=\left\{E_{1},\cdots ,E_{K}\right\}$ and vertices $Vertices\left(DT\left(Q\right)\right)=\left\{V_{1},\cdots ,V_{K}\right\}$ based on the triangulation to a graph-based clustering technique as an undirected network. Here, K is 6N−6 at maximum. Applying Delaunay triangulation to simulated spatial data with seven clusters, as shown in Figure 4a, a graph is obtained, as seen in Figure 4b. As a result of segmentation, the triangles located at the outer periphery of the cluster or located between different clusters are formed as triangles with relatively wide or long nodes. The triangles inside the clusters, on the other hand, vary depending on the cluster density, but are generally dense so that the area is small or the edges are short. In this case, triangulation further removes the unnecessary components constituting the graph after taking the standard score of the area and the length of edges of the divided triangle as parameters [30]. To do this, the mean and variance of not only the areas, $Area\left(DT\left(Q\right)\right)=\left\{A_{1},\cdots ,A_{H}\right\}$, but also the edge lengths, $Length\left(DT\left(Q\right)\right)=\left\{L_{1},\cdots ,L_{K}\right\}$, of all of the triangles are computed and applied to Equations (2)–(3).

This section may be divided by subheadings. It should provide a concise and precise description of the experimental results, their interpretation as well as the experimental conclusions that can be drawn.

(2) $$Area_{Z}=\frac{Area_{mean}\left(DT\left(Q\right)\right)-Area\left(DT\left(Q\right)\right)}{\left(Area_{var}\left(DT\left(Q\right)\right)\right)}$$

(3) $$Length_{Z}=\frac{Length_{mean}\left(DT\left(Q\right)\right)-Length\left(DT\left(Q\right)\right)}{Length_{var}\left(DT\left(Q\right)\right)}$$

Finally, after eliminating unnecessary node components from the optimal value for the data distribution characteristic, we have the graph shown in Figure 4c. It can be seen that some noise components existing in the data have been removed by a threshold based on a standard score calculated from Equations (2) and (3), however, the touching problem and chain noise still remain to some degree. These points are locally formed with a wide triangle or a long side, and have a low density with a small number of connected nodes compared to other points.

The proposed DTSCAN utilizes the existing DBSCAN clustering mechanism to form clusters by calculating the density with neighbor nodes based on the spatial density to remove these connection components and detect clusters without loss of data. DBSCAN is a clustering method based on the density of neighboring data, unlike clustering based on the data distribution. It is suitable for processing spatial data, including noise, and can distinguish clusters of various shapes and sizes. The graph-based DTSCAN does not take into account the minimum adjacent radius of the DBSCAN parameters. However, only a minimum number of neighboring nodes, MinPts, is defined to search neighboring nodes connected to themselves around an arbitrary node inside the graph, which is regarded as a cluster. DTSCAN is designed to form a cluster if the number of neighboring nodes is more than MinPts and to perform the same tests around adjacent nodes to extend the cluster. The clustering process of DTSCAN is summarized as follows:

A neighboring node of point $p_{i}$ in a triangle is a set of edge graphs directly connected to $p_{i}$.
If the number of elements in the set of connected neighboring nodes is greater than or equal to MinPts, it is included as one cluster, $C_{q}$.
(1)–(2) is repeated for neighboring nodes of point $p_{i}$ and the cluster is expanded by searching for neighboring nodes.
Points without connection nodes are classified as noise or anomalous points.

**Figure 5:**
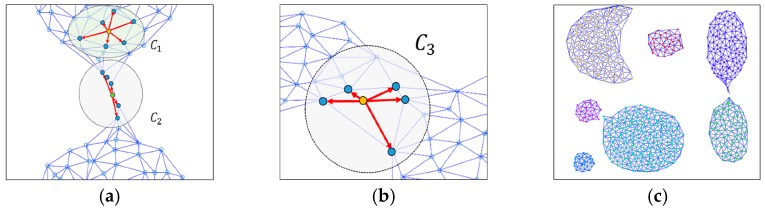
Touching problems of simulated data and their solution through DTSCAN. (**a**,**b**) enlarged illustrations of Figure 4c; (**c**) the clustering result via DTSCAN.

The aim of this study is to overcome the limitations of existing clustering techniques in a complex spatial data space and to devise a more advanced spatial clustering technique, especially considering touching problems. Assuming that MinPts is 6 in the illustrated example of Figure 4, $C_{1}$ is included in a certain cluster, but $C_{2}$ and $C_{3}$ do not satisfy the MinPts condition and are moved away from the cluster, as shown in Figure 5a,b, such that we can obtain improved clustering implementation results through the proposed DTSCAN, as shown in Figure 5c.

## 4. Experimental Results

To evaluate the performance of the proposed DTSCAN method, three sets of virtual two-dimensional spatial data and three-dimensional point clouds collected from a LiDAR mounted on an actual terrestrial vehicle were utilized [31,32,33]. Two-dimensional spatial data were selected as a data set including uneven density characteristics, various cluster shapes, and noise, and the images included in the KITTI benchmark-object detection were used as point clouds, where the pedestrians assigned to the class were selected on an object-by-object basis [34].

The purpose of spatial data clustering is to divide the whole into a meaningful group using distance or another similarity metric. To evaluate the performance of the proposed clustering technique, we employed the concept of the point score range (PSR), used as an index of 3D spatial data clustering performance in [35]—this value represents the degree of similarity between clusters with an intersection ratio, and the similarity for the jth cluster is expressed by Equation (4).

(4) $$PSR\left(j\right)=\max_{i\in 1,\ldots ,M}\frac{O_{j}\cap C_{i}}{\left(O_{j}\cup C_{i}-\left(O_{j}\cap C_{i}\right)\right)}$$

where $O_{j}$ is an index of a pre-assigned jth data set, and $C_{i}$ is an index value of data belonging to an ith cluster derived through DTSCAN. When all clusters are compared in a one-to-one correspondence, the set with the highest PSR is defined as the *j*th cluster. In addition, we used the variation score range (VSR) defined by Equation (5) with the variance of clusters derived from the PSR for cluster similarity analysis.

(5) $$PSR\left(j\right)=\max_{i\in 1,\ldots ,M}\frac{O_{j}\cap C_{i}}{\left(O_{j}\cup C_{i}-\left(O_{j}\cap C_{i}\right)\right)}$$

To compare the performance of DTSCAN, five clustering techniques actively used in spatial data mining were adopted. They are k-means based on Euclidean distance, CURE, which is a hierarchical clustering scheme, EM [36] using the Gaussian probability distribution model, DBSCAN on the basis of the analysis of the data density between neighbors, and SC, which is a graph-based technique. K-means, CURE, and EM, which require a pre-cluster number, were actively searched by applying the Elbow [37] method used for cluster verification and analysis, and the internal parameters of CURE, DBSCAN, and SC were selected and optimized for the evaluation data.

### 4.1. Performance Evaluation with 2D Synthetic Data

**Figure 6:**
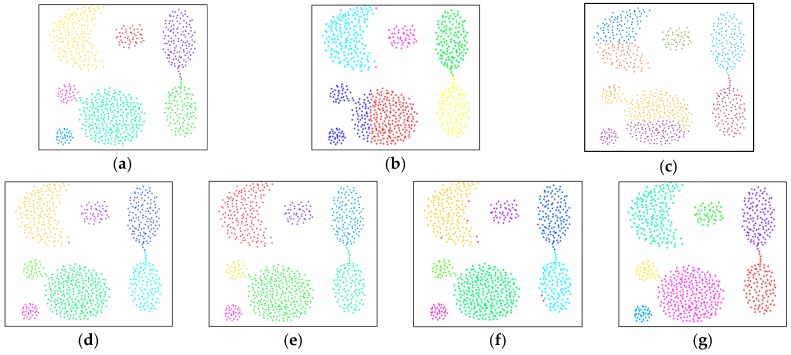
Clustering performance comparisons using a data set, S1. (**a**) original data set (**b**) K-means, (**c**) EM, (**d**) DBSCAN, (**e**) CURE, (**f**) SC, and (**g**) DTSCAN.

The performance of the proposed spatial clustering methodology was first evaluated based on a dataset with three types of 2D simulation, S={S1, S2, S3}. Figure 6a shows synthetic data set *S1* according to the pre-assigned cluster information, and can confirm complicated spatial data, including data sets experiencing the contact problem and nonlinearity. The performance comparison results between the proposed DTSCAN and other clustering schemes are summarized in Table 1 and demonstrated in Figure 6b–f. The proposed DTSCAN showed superior clustering performance compared to other clustering schemes, and successfully classifies all seven clusters in S1 data. Since only one data point was not included in any cluster, the average of the total PSR and VSR was as high as 0.999. In the case of k-means and EM, clusters close to a circle shape were classified relatively successfully, but clusters with a nonlinear shape, or in which contact problems exist, were not classified, so that one cluster divided into several or adjacent clusters was considered as one single cluster.

**Figure 7:**
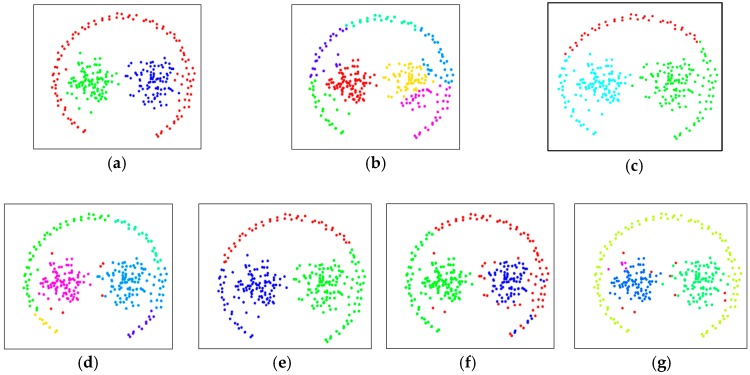
Clustering performance comparisons using a data set, S2. (**a**) original data set, (**b**) K-means, (**c**) EM, (**d**) DBSCAN, (**e**) CURE, (**f**) SC, and (**g**) DTSCAN.

As a result, the PSR was a low value and the VSR was a value somewhat far from 1. On the other hand, DBSCAN, CURE and SC succeeded in categorizing all clusters at almost the same level as DTSCAN. However, very few data located outside the clusters were considered as noise and slight performance degradation occurred. Figure 7a shows data set S2 used for another performance evaluation according to the previously given cluster information. As shown, other clusters are included in one circular cluster; each cluster has different densities and can be defined as complex spatial data similar to S1. The test results comparing the proposed DTSCAN and other clustering schemes are summarized in Table 2 and are shown in Figure 7b–f. The results shown in the test evaluation based on the S2 data set were very remarkable. DTSCAN was the only one that successfully classified all three clusters that other techniques could not classify. Some of the data with contact problems were regarded as noise and adversely affected clustering performance, but the PRS of DTSCAN was the highest and the VSR was very close to 1. For k-means, CURE, and EM, the PSR is less than 0.7 and the VSR is farther from 1 due to the limitations of a distance-based clustering architecture. In the case of DBSCAN, the shape of the cluster was implemented similar to DTSCAN so that the VSR was formed to be relatively close to 1, but the contact problem and the failure of the outermost section of the data cluster caused the PSR to be as low as 0.7.

**Figure 8:**
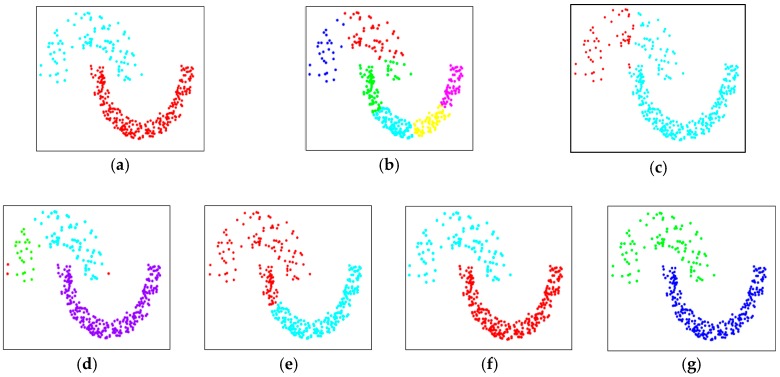
Clustering performance comparisons using a data set, S3. (**a**) original data set, (**b**) K-means, (**c**) EM, (**d**) DBSCAN, (**e**) CURE, (**f**) SC, and (**g**) DTSCAN.

Figure 8a shows the evaluation data, S3, in accordance with pre-assigned cluster information, which seems rather simple, but the two clusters are adjacent to each other and have a nonlinear shape. In addition, the density of data constituting each cluster is different from each other, and in particular, the density at the top of the cluster is very uneven. The performance evaluation results of the proposed DTSCAN and other clustering schemes are listed in Table 3 and illustrated in Figure 8b–f. As with the clustering results of S2 data, all other clustering techniques except DTSCAN and SC did not successfully find two clusters in the data. In the case of DBSCAN, the clusters at the bottom were successfully separated, but the upper clusters failed to cluster because of the uneven density inside. As a result of the performance evaluation using all these two-dimensional synthetic data, the superior performance of the proposed DTSCAN was demonstrated.

All experiments were performed based on Matlab 2019a / CPU: i7-7700, and the average operation speed of DBSCAN, SC, and DTSCAN was at 5ms, 70ms, and 700ms, respectively, in the tests using 2D synthetic data (S1, S2, S3). Preprocessing such as triangulation and global/local effect elimination need to be preceded, which requires a relatively high computational amount. Nevertheless, it would be very useful in clustering applications that require high noise robustness.

### 4.2. Performance Evaluation using 3D Point Clouds

To verify the applicability of the proposed DTSCAN in a real environment, we performed an additional performance evaluation of the object separation problem of 3D point clouds collected from LiDAR. Importantly, 3D point cloud data can be considered sparse data in Euclidean space and contain information about various terrains as well as objects. However, clusters representing the shape of objects are irregular and have arbitrary pattern features, which is an inherent limitation of LiDAR, making it more difficult to detect and isolate the boundaries of neighboring objects in the presence of various internal/external environmental factors [38].

**Figure 9:**
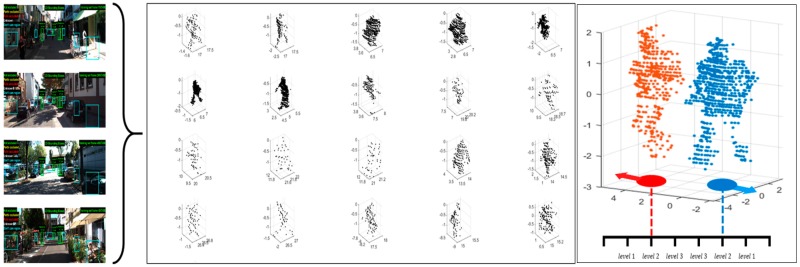
Pedestrian 3D point clouds extracted from KITTI dataset.

The KITTI vision benchmark suite used for performance verification is a set of data for computer vision research on autonomous driving, including pedestrians utilized in this study as well as various objects such as cars and trains. However, due to the limitations of the driving distance and parking safety distance, the distance between vehicles should be maintained at a minimum of 0.8–1 m. Because of this, it is observed with a specific pattern of ‘L’ or ‘I’ shape [39], so separation and adjacent boundary detection is relatively easy and has not been utilized in performance tests. For evaluation, 10 single objects within a detection distance of 15 m were randomly extracted from the data set. Then, two objects were arbitrarily selected and preprocessed in the form of zero mean and unit variance, and then divided into three levels so as to be placed close to each other. Figure 9 shows the 20 evaluation data created. The three proximity levels of the two objects were defined by shifting the mean value by a factor of λ, giving the change of the coordinate value with respect to the center point of the x-y plane of the object. The case where the distance between objects is the farthest is level 1 (λ = 4), and the closest case is level 3 (λ = 1).

**Figure 10:**
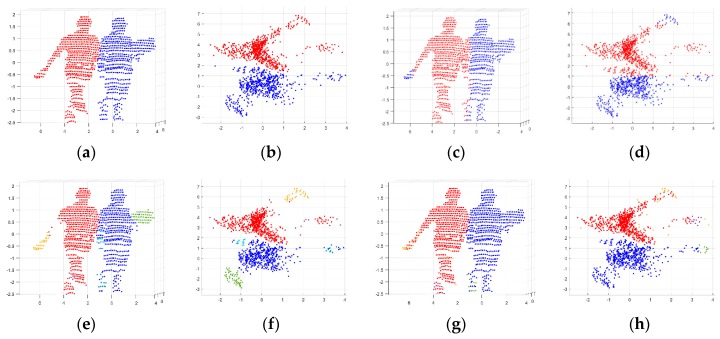
Clustering performance comparisons using 3D point clouds. (**a**,**b**) front view of the original data and its top view; (**c**,**d**) clustering results by SC; (**e**,**f**) clustering results by DBSCAN; (**g**,**h**) clustering results by DTSCAN.

Table 4 compares the performance evaluation results of the proposed DTSCAN and DBSCAN according to the three proximity levels of two persons, and Figure 10 shows some data used in the evaluation alongside their object separation results. Compared with DBSCAN, the proposed DTSCAN showed excellent performance irrespective of object distance according to the evaluation level. In particular, the higher the level, the lower the PSR due to contact problems, but the VSR representing the data distribution maintained close to 1. On the other hand, DBSCAN showed similar performance to DTSCAN when the evaluation level was low, but all indices were lowered as the distance between objects became lower, especially VSR, which was significantly lowered.

## 5. Conclusions

As the fields of machine learning and pattern recognition have grown steadily, space clustering techniques with superior performance have begun to emerge. These techniques have shown that not only clusters with uniform data distribution and an ideal shape, but also clusters with nonlinear appearance have excellent separation performance, and can be applied in various fields. However, some issues still need to be addressed for efficient spatial data clustering. In spatial data with complexity, different clusters can be very contiguous, and the density of each cluster can be arbitrary and uneven. In addition, background noise that does not belong to any cluster in the data or chain noise that connects multiple clusters may be included. These features make it more difficult to isolate adjacent clusters, so a new approach is required to solve the nonlinear shape, irregular density, and touching problems of adjacent clusters that are common in complex spatial data clustering, as well as to improve robustness against various types of noise in spatial clusters. We thus proposed an efficient way to cluster spatial data based on Delaunay triangulation and the mechanism of the existing DBSCAN. In the performance evaluation using simulated synthetic data as well as real 3D point clouds, the proposed technique maintained better clustering performance compared to other clustering techniques and detected all clusters in the data without loss. In particular, the experimental result using point clouds demonstrated that the proposed architecture is superior to DBSCAN in terms of the contact problem with adjacent clusters and the chain noise. In the near future, we plan to extend this technique to include non-spatial properties that can lead to in-depth analysis, and apply it to large-scale spatial data such as GPS or earthquakes, and to confirm the applicability of the proposed technique and develop it as a generalized clustering model.

## Figures and Tables

**Table 1:** Comparison of clustering performance using a data set, S1, in terms of PSR and VSR.

Clustering Scheme	K-Means	EM	DBSCAN	CURE	SC	DTSCAN
Avg. *PSR*	0.741	0.703	0.974	0.984	0.986	**0.999**
Avg. *VSR*	3.822	5.274	1.022	1.013	1.017	**0.999**

**Table 2:** Comparison of clustering performance using a data set, S2, in terms of PSR and VSR.

Clustering Scheme	K-Means	EM	DBSCAN	CURE	SC	DTSCAN
Avg. *PSR*	0.635	0.552	0.739	0.624	0.651	**0.934**
Avg. *VSR*	0.581	1.603	0.938	1.482	1.446	**0.959**

**Table 3:** Comparison of clustering performance using a data set, S3, in terms of PSR and VSR.

Clustering Scheme	K-Means	EM	DBSCAN	CURE	SC	DTSCAN
Avg. *PSR*	0.428	0.752	0.864	0.734	**1.000**	**1.000**
Avg. *VSR*	0.274	1.245	0.849	1.022	**1.000**	**1.000**

**Table 4:** Comparison of clustering performance using 3D point clouds in terms of PSR and VSR.

		SC	DBSCAN	DTSCAN
**Level 1**	Avg. *PSR*	0.97	0.96	**0.98**
	Avg. *VSR*	1.14	0.91	**0.96**
**Level 2**	Avg. *PSR*	0.86	0.87	**0.89**
	Avg. *VSR*	0.89	0.81	**0.93**
**Level 3**	Avg. *PSR*	0.69	0.68	**0.77**
	Avg. *VSR*	1.29	0.59	**0.89**
